# Supplementary material for: Determinants of transthyretin levels and their association with adverse clinical outcomes among UK Biobank participants
Source: Nat Commun. 2024 Jul 23;15:6221. doi: 10.1038/s41467-024-50231-1 (PMC11266646; doi:10.1038/s41467-024-50231-1)
Supplement: Supplementary file 1 — Supplementary Information [file 41467_2024_50231_MOESM1_ESM.pdf]

Supplementary Table 1: International Classification of Diseases, Ninth Revision and Tenth Revision, Clinical Modification Codes Used to Identify Study Outcomes

	ICD Codes
**Heart Failure**	I11.0,I13.0,I13.2,I25.5,I42.0,I42.5,I42.8,I42.9,I50,I50.0,I50.1,I50.9,402.01,402.11,402.91,404.01,404.03,404.11,404.13,404.91,404.93,428,428.0,428.1,428.3,428.4,428.9,425.4
**Coronary Artery Disease**	I21,I21.0,I21.1,I21.2,I21.3,I21.4,I21.9,I22,I22.0,I22.1,I22.8,I22.9,I23,I23.0,I23.1,I23.2,I23.3,I23.4,I23.5,I23.6,I23.8,I24,I24.0,I24.1,I24.8,I24.9,I25.2,410,410.9,411,411.9,412,412.9 K40,K40.1,K40.2,K40.3,K40.4,K40.8,K40.9,K41,K41.1,K41.2,K41.3,K41.4,K41.8,K41.9,K42,K42.1,K42.2,K42.3,K42.4,K42.8,K42.9,K43,K43.1,K43.2,K43.3,K43.4,K43.8,K43.9,K44,K44.1,K44.2,K44.8,K44.9,K45.1,K45.2,K45.3,K45.4,K45.5,K45.6,K45.8,K45.9,K46,K46.1,K46.2,K46.3,K46.4,K46.5,K46.8,K46.9,K49.1,K49.2,K49.3,K49.4,K49.8,K49.9,K50.1,K50.2,K50.4,K75.1,K75.2,K75.3,K75.4,K75.8,K75.9
**Stroke**	G45,G45.0,G45.1,G45.3,G45.4,G45.8,G45.9,G46,G46.3,G46.4,G46.5,G46.7,G46.8,I63,I63.0,I63.2,I63.3,I63.5,I63.8,I63.9,I64,I65,I65.0,I65.1,I65.2,I65.3,I65.8,I65.9,I66,I66.0,I66.1,I66.2,I66.3,I66.4,I66.8,I66.9,I67.2,I69.4,433,434,433.1,433.9,434.9,435.9,436.9,437.0,437.1,437.8,437.9,438.9
