# Supplementary material for: Determinants of transthyretin levels and their association with adverse clinical outcomes among UK Biobank participants
Source: Nat Commun. 2024 Jul 23;15:6221. doi: 10.1038/s41467-024-50231-1 (PMC11266646; doi:10.1038/s41467-024-50231-1)
Supplement: Supplementary file 3 — Reporting Summary [file 41467_2024_50231_MOESM3_ESM.pdf]

nature portfolio

Corresponding author(s): Pankaj Arora

Last updated by author(s): Apr 30, 2024

Reporting Summary

Nature Portfolio wishes to improve the reproducibility of the work that we publish. This form provides structure for consistency and transparency in reporting. For further information on Nature Portfolio policies, see our Editorial Policies and the Editorial Policy Checklist.

Statistics

For all statistical analyses, confirm that the following items are present in the figure legend, table legend, main text, or Methods section.

n/a	Confirmed	
☐	☒	The exact sample size (*n*) for each experimental group/condition, given as a discrete number and unit of measurement
☐	☒	A statement on whether measurements were taken from distinct samples or whether the same sample was measured repeatedly
☐	☒	The statistical test(s) used AND whether they are one- or two-sided<br>*Only common tests should be described solely by name; describe more complex techniques in the Methods section.*
☐	☒	A description of all covariates tested
☒	☐	A description of any assumptions or corrections, such as tests of normality and adjustment for multiple comparisons
☐	☒	A full description of the statistical parameters including central tendency (e.g. means) or other basic estimates (e.g. regression coefficient) AND variation (e.g. standard deviation) or associated estimates of uncertainty (e.g. confidence intervals)
☐	☒	For null hypothesis testing, the test statistic (e.g. *F*, *t*, *r*) with confidence intervals, effect sizes, degrees of freedom and *P* value noted<br>*Give P values as exact values whenever suitable.*
☒	☐	For Bayesian analysis, information on the choice of priors and Markov chain Monte Carlo settings
☒	☐	For hierarchical and complex designs, identification of the appropriate level for tests and full reporting of outcomes
☒	☐	Estimates of effect sizes (e.g. Cohen's *d*, Pearson's *r*), indicating how they were calculated

Our web collection on statistics for biologists contains articles on many of the points above.

Software and code

Policy information about availability of computer code

Data collection	Data was collected by the UK Biobank.
Data analysis	All analysis was conducted on SAS 9.4 (Cary, NC).

For manuscripts utilizing custom algorithms or software that are central to the research but not yet described in published literature, software must be made available to editors and reviewers. We strongly encourage code deposition in a community repository (e.g. GitHub). See the Nature Portfolio guidelines for submitting code & software for further information.

Data

Policy information about availability of data

All manuscripts must include a data availability statement. This statement should provide the following information, where applicable:

- Accession codes, unique identifiers, or web links for publicly available datasets
- A description of any restrictions on data availability
- For clinical datasets or third party data, please ensure that the statement adheres to our policy

Data in the UK Biobank (www.ukbiobank.ac.uk) are available to researchers with an approved application. This study was conducted under UK Biobank Application Number 58838.

Research involving human participants, their data, or biological material

Policy information about studies with human participants or human data. See also policy information about sex, gender (identity/presentation), and sexual orientation and race, ethnicity and racism.

Reporting on sex and gender	This study used sex reported in UK Biobank Data Field 31 to define baseline characteristics and as a covariate in the statistical models.
Reporting on race, ethnicity, or other socially relevant groupings	Race and ethnicity based analysis were not performed.
Population characteristics	This study included 35,206 individuals. The median age was 558 (50, 63) years and 19,399 (55.1%) females.
Recruitment	Participants between the age of 40 and 69 years were recruited from 22 assessment centers across the United Kingdom.
Ethics oversight	The UK Biobank received ethical approval from the North West Multicenter Research Ethical Committee.

Note that full information on the approval of the study protocol must also be provided in the manuscript.

Field-specific reporting

Please select the one below that is the best fit for your research. If you are not sure, read the appropriate sections before making your selection.

☒ Life sciences ☐ Behavioural & social sciences ☐ Ecological, evolutionary & environmental sciences

For a reference copy of the document with all sections, see nature.com/documents/nr-reporting-summary-flat.pdf

Life sciences study design

All studies must disclose on these points even when the disclosure is negative.

Sample size	35,206 individuals. No sample size calculations for power were conducted. All individuals enrolled in the UK Biobank were included.
Data exclusions	Individuals of lacking genetic and proteomic data, related individuals, individuals who did not consent to provide access to their electronic health records, individuals with prevalent cardiovascular disease, individuals with missing data on covariates, and pregnant females.
Replication	The current study was performed using data from the UK Biobank. Replication was not performed due to the lack of a comparable dataset.
Randomization	Given the observational cohort study design of the current analysis, randomization was not relevant. Instead of randomization, multivariable adjusted Cox models were used to account for clinically relevant covariates.
Blinding	This study is observational cohort study and did not include a specific intervention and had pre-collected data on outcomes. Therefore, blinding was not relevant in the current study.

Reporting for specific materials, systems and methods

We require information from authors about some types of materials, experimental systems and methods used in many studies. Here, indicate whether each material, system or method listed is relevant to your study. If you are not sure if a list item applies to your research, read the appropriate section before selecting a response.

Materials & experimental systems

n/a	Involved in the study	
☒	☐	Antibodies
☒	☐	Eukaryotic cell lines
☒	☐	Palaeontology and archaeology
☒	☐	Animals and other organisms
☒	☐	Clinical data
☒	☐	Dual use research of concern
☒	☐	Plants

Methods

n/a	Involved in the study	
☒	☐	ChIP-seq
☒	☐	Flow cytometry
☒	☐	MRI-based neuroimaging
